# Supplementary material for: Gene Polymorphism of Biotransformation Enzymes and Ciprofloxacin Pharmacokinetics in Pediatric Patients with Cystic Fibrosis
Source: Biomedicines. 2022 May 2;10(5):1050. doi: 10.3390/biomedicines10051050 (PMC9139162; doi:10.3390/biomedicines10051050)
Supplement: Supplementary file 1 [file biomedicines-10-01050-s001.zip › biomedicines-1683221-supplementary.pdf]

Supplement

Table S1. Parameters of physical development of patients depending on CYP2C9*3 gene polymorphism (I359L, c.1075A>C).

Parameter	Statistical indicators	Genotype		
		AA	AC	CC
Weight, kg	N	25	8	1
	Mean (±SD)	27.82 (13.05)	32.3 (14.70)	51
	Median (75%Q3-25%Q1)	24.5 (32.8-18.0)	35.25 (43.70-20.25)	51
	CV	47.03	45.5	-
Height, cm	N	25	8	1
	Mean (±SD)	127.49 (22.45)	130 (28.63)	166
	Median (75%Q3-25%Q1)	125(142–112)	137.75 (155–110)	-
	CV	17.61	21.91	-
BMI, kg/m^2	N	25	8	1
	Mean (±SD)	16.16 (2.30)	17.64 (2.78)	18.5
	Median (75%Q3-25%Q1)	15.4 (18.0–14.3)	16.75 (18.65–15, I)	18.5
BMI percentile	N	25	8	1
	Mean (±SD)	39.67 (29.79)	51.62 (33.35)	70.1
	Median (75%Q3-25%Q1)	32.20 (61.3–14.7)	51.35 (78.60–22.20)	-
	CV	75.09	64.60	-

Table S2. Parameters of physical development in groups of patients with different genotypes of CYP2D6*4 gene polymorphism (1846G>A).

Indicator	Parameter	**CYP2D6*4(1846G>A)**		
		GG	GA	AA
Weight, kg	N	19	14	1
	Mean (±SD)	29.21 (13.50)	30.79 (14.68)	19,0
	Median (75%Q3-25%Q1)	25.5 (38.5–18.0)	29.4 (46.0–15.2)	19,0
	CV	46.23	47.66	
Height, cm	N	19	14	1
	Mean (±SD)	129.88 (23.10)	129.75 (26.93)	114
	Median (75%Q3-25%Q1)	129.0 (143–117)	139.75 (154.5–97.0)	114
	CV	17.79	20.76	
BMI, kg/m^2	N	19	14	1
	Mean (±SD)	16.31 (2.27)	17.09 (2.73)	14.6
	Median (75%Q3-25%Q1)	16,4 (18,1-14,3)	16.35 (19.3–15.40)	14.6
	CV	13.94	15.96	
BMI percentile	N	19	14	1
	Mean (±SD)	42.8 (31,42)	44.96 (31,32)	32.2
	Median (75%Q3-25%Q1)	32.80 (70.1-14.7)	43.20 (65.5-13.2)	32.2
	CV	73.41	69.65	

Table S3. Indicators of physical development of children with different genotypes of GSTP1 gene polymorphism.

Indicator	Statistical indicators	Genotypes of *GSTP1*		
		AA	AG	GG
Weight, kg	N	15	16	2
	Mean (±SD)	29.09 (15.29)	28.35 (11.70)	32 (19.80)
	Median (75%Q3-25%Q1)	25.0 (46.0–15.2)	26.0 (35.75–19.25)	32.0 (46–18)
	CV	52.54	41.28	61.87
Height, cm	N	15	16	2
	Mean (±SD)	126.81 (28.48)	128.97 (19.24)	133.25 (30.05)
	Median (75%Q3-25%Q1)	129.0 (154–97)	128.5 (141.25–115.50)	133.25 (154.50–112.0)
	CV	22.45	14.92	22.55
BMI, kg/m^2	N	15	16	2
	Mean (±SD)	16.73 (2.40)	16.28 (2.58)	16.8 (3.54)
	Median (75%Q3-25%Q1)	16.40 (18.5–14, I)	15.80 (16.95–14.55)	16.8 (19.3–14.3)
	CV	14.32	15.86	21.04
BMI percentile	N	15	16	2
	Mean (±SD)	46.83 (33.23)	38.18 (29.55)	45.65 (28.07)
	Median (75%Q3-25%Q1)	47.2 (77.3–14.7)	32.05 (54.05–12.0)	45.65 (65.50–25.80)
	CV	70.96	77.38	61.49

Table S4. Indicators of physical development of children with different genotypes of the GCLC gene.

Indicator	Statistical indicators	GCLC genotype	
		other	7/7
Weight, kg	N	18	14
	Mean (±SD)	26.71 (11.67)	33.16 (15.35)
	Median (75%Q3-25%Q1)	23.80 (32.8–19.0)	29.50 (50.0–18.0)
	CV	43.68	46.29
Height, cm	N	18	14
	Mean (±SD)	126.61 (21.49)	133.59 (26.60)
	Median (75%Q3-25%Q1)	126.0 (142–110)	136.75 (155.5–117.0)
	CV	16.97	19.91
BMI	N	18	14
	Mean (±SD)	15.84 (1.81)	17.41 (2.97)
	Median (75%Q3-25%Q1)	15.80 (16.4–14.5)	17.8 (18.5–14.9)
	CV	11.40	17.05
BMI percentile	N	18	14
	Mean (±SD)	36.02(26.69)	51.55 (35.25)
	Median (75%Q3-25%Q1)	30.30 (52.8–13.2)	49.10 (91.7–25.8)
	CV	74.11	68.39

Table S5. Physical development indicators of children with different NAT2(341T>C) genotype variants.

Indicator	Statistical indicators	NAT2(341T>C)		
		TT	TC	CC
Age, years	N	7	15	12
	Mean (±SD)	9.0±3.40	10.13±4.78	6.83± 3.66
	Median (75%Q3-25%Q1)	9.0 (8.0)	10 (8.0)	6.5 (7.0)
	CV	48.86	47.16	53.62
Weight, kg	Mean (±SD)	33.61±14.96	32.03±14.12	24.11±11.75
	Median (75%Q3-25%Q1)	31.5 (31.4)	28.5 (28.6)	21.05 (12.95)
	CV	44.51	44.10	48.73
Height, cm	Mean (±SD)	133.96±24,73	135.17 (23.59)	119.42±23.31
	Median (75%Q3-25%Q1)	137 (47.7)	135.0 (41.5)	119 (41.25)
	CV	18.46	17.45	19.52
BMI, kg/m^2	Mean (±SD)	17.69± 2.13	16.48 ±2.23	16.05±2.85
	Median (75%Q3-25%Q1)	16.8 (2.7)	15.4 (3.9)	15.6 (3.05)
	CV	12.07	13.56	17.75
BMI percentile	Mean (±SD)	62.9±19.84	75±23,91	42.77±38.76
	Median (75%Q3-25%Q1)	66.9 (26.5)	32.2 (30.10)	29.6 (75.76)
	CV	31.54	68.8	90.63

Table S6. Pharmacokinetic parameters of CPF in children with different NAT2(341T>C) genotype variants.

Pharmacokinetic parameter	Statistical indicators	*NAT2*(341T>C)		
		TT	TC	CC
C_{max}, µg/ml	N	7	14	11
	Mean(±SD)	19.49±7.94	27.59±8.54	24.99 ±14.3
	Median (75%Q3-25%Q1)	17.89(5.22)	26.2 (11.11)	23.2 (21.81)
	CV	40.75	30.93	57.25
AUC_{0-t}, µg*h/ml	Mean(±SD)	66.11±18.48	104.12±37.94	81.02±49.37
	Median (75%Q3-25%Q1)	74.60 (27.25)	100.75 (49.52)	61.79 (76.80)
	CV	27.95	36.44	60.94
T_{max}, hours	Mean(±SD)	2.36±1.46	2.35±1.28	2.45±1.01
	Median (75%Q3-25%Q1)	1.5 (3.0)	1.5 (1.5)	3.0 (1.5)
	CV	62.10	54.19	41.20
AUC_{0-t_norm}, (µg*h/ml) / (mg/kg)	Mean(±SD)	3.27±0,93	4.82±1.95	3.69±2.08
	Median (75%Q3-25%Q1)	3.31 (1.81)	4.16 (3.11)	3.09 (2.73)
	CV	28.54	40.37	56.39
C_{max_norm}, (µg/ml)/(mg/kg)	Mean(±SD)	0.94±0.27	1.26±0,39	1.13 (0.57)
	Median (75%Q3-25%Q1)	0.96 (0.32)	1.31 (0.49)	1.20 (0.96)
	CV	29.12	30.79	50.07

Table S7. Evaluation of logistic regression parameters in patients aged 6 to 16 years with different variants of the TT, TC, CC genotypes of the genetic variation of NAT2 (341T>C).

Predictor	Regression coefficient	Standardized regression coefficient	Wald Chi^2	The level of statistical significance, p	Standard Error
TT genotype	64.5976		4.0616	0.0439	32.0531
TS genotype	69.3073		4.4274	0.0354	32.9386
delF508/delF508 genotype	4.6097	1.1241	5.6348	0.0176	1.9419
Concentration at point 1, µg/ml	-0.6914	-4.8612	6.0248	0.0141	0.2817
Concentration at point 2, µg/ml	-1.0282	-6.8763	6.0623	0.0138	0.4176
Concentration at point 5, µg/ml	-1.1881	-3.2045	5.1299	0.0235	0.5246
C_{max}, µg/ml	3.8207	21.5040	4.8007	0.0284	1.7438
T_{max}, hours	-2.8200	-1.9753	4.9778	0.0257	1.2640
AUC_{0-t_norm}, (µg*h/ml) / (mg/kg)	10.4778	10.6415	6.4647	0.0110	4.1209
C_{max_norm}, (µg/ml)/(mg/kg)	-88.4679	-20.0454	5.0627	0.0244	39.3185
Dose of CPF, mg/kg	-2.8292	-4.8476	4.0365	0.0445	1.4082

Table S8. Demographic indicators and parameters of physical development of children and adolescents with the F508del/F508del genotype and "other" genotypes.

Indicator	Statistical indicators	Genotype	
		"Other"	F508del/ F508del
Age, years	N	25	10
	Mean (±SD)	9.08(4.43)	8.6(5.10)
	Median (75%Q3-25%Q1)	8 (13–6)	9.5 (12–3)
	CV	48.83	59.34
Weight, kg	Mean (±SD)	30.13 (13.92)	31.57 (17.33)
	Median (75%Q3-25%Q1)	26 (46–19)	28.5 (41.4–15.2)
	CV	46.19	54.90
Height, cm	Mean (±SD)	131.00 (22.29)	129.3 (31.51)
	Median (75%Q3-25%Q1)	129(154–114)	136 (155.5–94.5)
	CV	17.02	24.37
BMI, kg/m^2	Mean (±SD)	16.50 (2.70)	17.33 (2.31)
	Median (75%Q3-25%Q1)	16.20 (18.1–14.5)	17.35 (18.50–15.40)
	CV	16.37	13.35
BMI percentile	Mean (±SD)	40.41 (28.32)	53 (34.59)
	Median (75%Q3-25%Q1)	32.80 (63.3–14.7)	52.40 (90.5–28.70)
	CV	70.09	65.27

Table S9. Pharmacokinetic parameters of CPF in children and adolescents with the F508del/F508del genotype and other genotypes.

Parameter	Statistical indicators	Genotypes	
		"Other"	delF508/delF508
Dose, mg/kg	N	24	9
	Mean (±SD)	C21.8 (3.38)	20.9 (2.80)
	Median (75%Q3-25%Q1)	20.40 (24.75-19.50)	20.00 (24.0-19.5)
	CV	15.52	13.39
Concentration at point 1, μg/ml	Mean (±SD)	18.43 (13.99)	13.93 (8.90)
	Median (75%Q3-25%Q1)	17.51 (29.95-6.24)	17.31 (19.79-5.40)
	CV	75.86	63.92
Concentration at point 2, μg/ml	Mean (±SD)	17.67(12.68)	15.49 (7.15)
	Median (75%Q3-25%Q1)	12.83 (25.20-8.50)	12.20 (19.20-11.10)
	CV	71.74	46.20
Concentration at point 3, μg/ml	Mean (±SD)	14.11 (9.51)	11.32 (5.41)
	Median (75%Q3-25%Q1)	11.71 (22.87-11.71)	10.23 (14.70-7.02)
	CV	67.37	47.80
Concentration at point 5, μg/ml	Mean (±SD)	6.03 (5.04)	4.37 (3.27)
	Median (75%Q3-25%Q1)	4.35 (8.95-1.93)	4.30 (5.27-1.90)
	CV	83.59	74.92
AUC_{0-t}, μg*h/ml	Mean(±SD)	91.30 (45.19)	74.39 (25.71)
	Median (75%Q3-25%Q1)	77.64 (114.50-65.14)	63.78 (80.73-61.17)
	CV	49.49	34.56
C_{max}, μg/ml	Mean(±SD)	26.20 (12.11)	19.94 (5.62)
	Median (75%Q3-25%Q1)	24.84 (33.31-17.91)	19.79 (21.40-17.31)
	CV	46.20	28.20
T_{max}, hours	Mean(±SD)	2.44 (1.24)	2.5 (1.30)
	Median (75%Q3-25%Q1)	1.50 (3.0-1.5)	1.50 (3.0-1.5)
	CV	50.72	51.96
AUC_{0-t_norm}, (μg*h/ml) / (mg/kg)	Mean(±SD)	4.18 (1.98)	3.68 (1.67)
	Median (75%Q3-25%Q1)	3.83 (5.28-2.99)	3.27 (4.04-2.52)
	CV	47.34	45.51
C_{max_norm}, (μg/ml) / (mg/kg)	Mean(±SD)	1.18 (0.47)	0.99 (0.39)
	Median (75%Q3-25%Q1)	1.21 (1.44-0.95)	0.83 (1.07-0.75)
	CV	39.38	39.84
